# Baboon induced pluripotent stem cell generation by *piggyBac* transposition of reprogramming factors

**DOI:** 10.5194/pb-6-75-2019

**Published:** 2019-07-29

**Authors:** Ignacio Rodriguez-Polo, Michael Stauske, Alexander Becker, Iris Bartels, Ralf Dressel, Rüdiger Behr

**Affiliations:** 1 Research Platform Degenerative Diseases, German Primate Center – Leibniz Institute for Primate Research, Kellnerweg 4, 37077 Göttingen, Germany; 2 German Center for Cardiovascular Research (DZHK), Partner site, Göttingen, Germany; 3 Institute of Human Genetics, University Medical Center Göttingen, Robert-Koch-Str. 40, 37075 Göttingen, Germany; 4 Institute of Cellular and Molecular Immunology, University Medical Center Göttingen, Robert-Koch-Str. 40, 37075 Göttingen, Germany; a current address: BlueRock Therapeutics, 101 College St, PMCRT 14-301, Toronto, ON M5G 1L7, Canada

## Abstract

Clinical application of regenerative therapies using embryonic or induced pluripotent stem cells is within reach. Progress made during recent years has encouraged researchers to address remaining open questions in order to finally translate experimental cell replacement therapies into application in patients. To achieve this, studies in translationally relevant animal models are required to make the final step to the clinic. In this context, the baboon (*Papio anubis*) may represent a valuable nonhuman primate (NHP) model to test cell replacement therapies because of its close evolutionary relationship to humans and its large body size. In this study, we describe the reprogramming of adult baboon skin fibroblasts using the *piggyBac* transposon system. Via transposon-mediated overexpression of six reprogramming factors, we generated five baboon induced pluripotent stem cell (iPSC) lines. The iPSC lines were characterized with respect to alkaline phosphatase activity, pluripotency factor expression analysis, teratoma formation potential, and karyotype. Furthermore, after initial cocultivation with mouse embryonic fibroblasts, we were able to adapt iPSC lines to feeder-free conditions. In conclusion, we established a robust and efficient protocol for iPSC generation from adult baboon fibroblasts.

## 1 Introduction

The development of successful approaches for the induction of pluripotency in somatic cells to generate induced pluripotent stem cells (iPSCs) was ground-breaking (Takahashi and Yamanaka, 2006). Pluripotent stem cells (PSCs) are promising for the treatment of degenerative diseases, which are associated with the progressive loss of functional cells in the patient. Regarding therapeutic application, iPSCs overcame the practical and ethical difficulties associated with the use of embryonic stem cells (ESCs) for cell replacement therapies (CRTs) (Takahashi et al., 2007). One therapeutic approach is to transplant iPSC-derived immature tissue-specific cells, such as neuro- or cardio-progenitor cells (Grow et al., 2016b). An alternative approach is the use of in vitro grown tissue, such as engineered heart muscle, generated from iPSC-derived cardiomyocytes cultured in three-dimensional tissue scaffolds (Tiburcy et al., 2017; Stevens et al., 2009; Turnbull et al., 2014).

The combined efforts of researchers resulted in a broad panel of reprogramming methods for the generation of iPSCs (Malik and Mahendra, 2013; Patel and Yang, 2010). This allows the exploration of different strategies for cell reprogramming, also impacting potential CRTs in humans (Sosa et al., 2017; Zhang et al., 2017). However, several aspects need to be addressed before routine clinical application including (i) long-term safety, (ii) survival and prevalence of the transplanted cells in the in vivo context of an immune system similar to humans, (iii) cell functionality after transplantation, and (iv) feasibility of the upscaling of the production of the cells and the size of the transplant. To achieve this, relevant preclinical animal models are required (Turnbull et al., 2014; Kobayashi et al., 2012; Grow et al., 2016a; Kimbrel and Lanza, 2015).

Within the spectrum of animal models suitable for translational research, nonhuman primates (NHPs) exhibit distinct advantages (Phillips et al., 2014; Behr, 2015). The similarities between humans and NHPs such as their genetic background, immune system, and anatomy make them an attractive model for the validation of new therapeutic strategies like CRTs (Grow et al., 2016a). Invasive biomedical research with great apes as the evolutionarily closest relatives of humans is restricted for ethical and legal reasons. Therefore and because of its relatively broad availability, rhesus macaques (*Macaca mulatta*) have been the NHP model of choice for many biomedical studies including neuroscience and HIV and AIDS research (Grow et al., 2016a; Navara et al., 2013; Didier et al., 2016). Although the rhesus macaque is a well-established model, the establishment of alternative models that share the advantageous characteristics of the rhesus macaque and exhibit specific additional advantages is desirable. The olive baboon (*Papio anubis*) is an Old World monkey with comparable evolutionary distance to humans as the macaques. It shares 92 % of the genome with humans, has a life expectancy of up to 45 years, and very closely resembles human development, anatomy, and physiology (Navara et al., 2018; Bailey, 2009; Rogers and Hixson, 1997). In comparison to macaques and other NHP species except for great apes, baboons have (i) a more similar size and weight in comparison with humans (16.4–21.3 kg for males and 10–15 kg for females) in contrast to rhesus (4–14.1 kg for males and 3–10 kg for females); (ii) they alternate quadrupedal and bipedal locomotion, and (iii) regarding transplantation studies, it is of relevance that their immune system closely resembles the human one, e.g., presenting the same four immunoglobulin G (IgG) subclasses (Grow et al., 2016a; Navara et al., 2018; Varilly and Chandler, 2008; Shearer et al., 1999). Furthermore, controlled-breeding colonies exist that have been studied for many years. Overall, baboons are an excellent NHP model for stem-cell-based transplantation studies (Navara et al., 2013; Bailey, 2009; Simerly et al., 2009; Agrba et al., 2016; Cox et al., 2013; Längin et al., 2018).

In parallel to addressing the remaining open questions of CRTs in animal models, the last years have seen an evolution of reprogramming approaches for the generation clinical grade iPSC (Malik and Mahendra, 2013; Patel and Yang, 2010; Kimbrel and Lanza, 2015). Virus-based reprogramming is very robust and efficient but poses a drawback for clinical application. The original retroviral reprogramming approach is associated with the proviral integration of the vector into the host cell genome and an increased oncogenic potential that is incompatible with the clinical use of respective iPSCs (Kimbrel and Lanza, 2015; Kim et al., 2012). Therefore, non-integrative (non-mutagenic) reprogramming approaches have been developed for human and mouse cells (Malik and Mahendra, 2013; Yu et al., 2009; Yamanaka et al., 2011). Recently, some of them have been adapted to macaques. For example, rhesus macaque iPSCs were generated from embryonic fibroblasts using self-replicating RNA engineered from the Venezuelan equine encephalitis (VEE) virus, which has a positive-strand RNA genome (Sosa et al., 2017). Also, episomal vectors have been used to generate iPSCs from macaque postnatal ear skin fibroblasts (Zhang et al., 2017). However, in both cases, this was achieved under feeder-dependent conditions. To test CRT preclinically in NHP, it is required to cultivate the cells under feeder-free conditions. Cultivation of iPSC using feeder cells, e.g., mouse embryonic fibroblasts (MEFs) or human foreskin fibroblasts (HFFs), limits mass-production and puts at risk the purity of the potential iPSC-derived graft (Villa-Diaz et al., 2013; Nishizawa et al., 2014; Hakala et al., 2009). Furthermore, it is important to have the option to generate vector-free iPSCs in order to preclinically test applications in humans (Shiba et al., 2016; Emborg et al., 2013; Wang et al., 2015).

In contrast to the macaque, the baboon has been widely neglected as an animal model for CRT testing, with very few exceptions (Simerly et al., 2009). Navarra and colleagues generated the first baboon iPSC (biPSC) using a retroviral approach (Navara et al., 2013). Recently this group also reprogrammed baboon peripheral blood cells using a non-integrating Sendai virus approach under feeder cell conditions (Navara et al., 2018). For thorough testing of the potential of CRT, however, it is necessary to have a broad panel of different cell lines available as inter iPSC line variability is common (Nishizawa et al., 2016; Carcamo-Orive et al., 2017; Ohi et al., 2011). Hence, it is important to generate different baboon iPSC lines by different reprogramming approaches and from different sources. This will allow the identification of the general and specific characteristics of baboon iPSC lines, which is important regarding the interpretation of preclinical data generated using NHPs and their translation into the clinics (Didier et al., 2016; Cox et al., 2013).

The *piggyBac* system has been shown to be useful for generating human, mouse, and marmoset monkey iPSCs (Debowski et al., 2015; Woltjen, 2011; Yusa et al., 2009; Mohseni et al., 2009). The *piggyBac* transposon-based approach represents several advantages such as (i) the robustness of the expression of the reprogramming factors, (ii) the capacity to deliver large DNA fragments, and (iii) the possibility of footprint-free excision of the vector after reprogramming. Using a six-factor-in-one vector transposon, we reported the successful reprogramming of marmoset monkey (*Callithrix jacchus*) postnatal skin fibroblasts into iPSCs (Debowski et al., 2015).

In this study, we adapted our established *piggyBac* reprogramming protocol to baboon cells in order to contribute to establishing this species as a preclinical model for CRTs. Employing the nonviral *piggyBac* approach, we were able to reproducibly reprogram baboon adult skin fibroblasts. We also managed to adapt baboon iPSC lines from feeder-dependent to feeder-free culture conditions, which will allow the xeno-free mass production of biPSCs.

## 2 Results

### 2.1 Baboon skin fibroblast reprogramming by the *piggyBac* transposition

**Figure 1:**
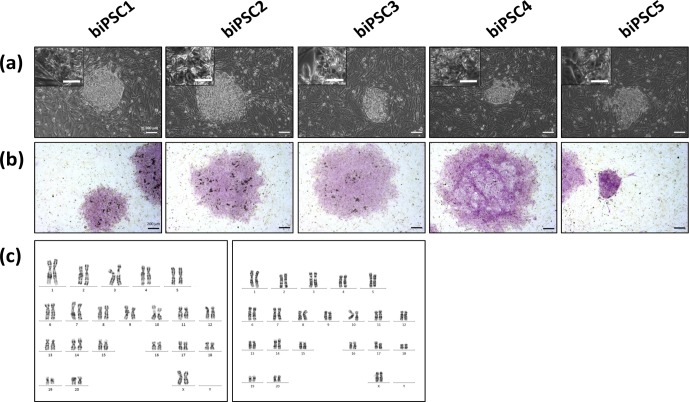
Baboon induced pluripotent stem cells. **(a)** iPSCs morphology of the five baboon lines generated and characterized (DPZ_biPSC1 to DPZ_biPSC5; scale bars 100 and 25  µm, inset). **(b)** Alkaline phosphatase staining (scale bar 200 µm). **(c)** Representative G-banded female karyotypes, female karyotypes (42; XX) of DPZ_biPSC1 (left karyogram) and DPZ_biPSC4 (right karyogram).

Five independent biPSC lines, termed DPZ_biPSC1 to DPZ_biPSC5, were derived from fibroblasts isolated from a skin biopsy of an adult female baboon. Reprogramming was performed using our previously published *piggyBac* six-factor transposon system, containing the marmoset *SOX2*, *OCT4*, *KLF4*, *c-MYC*, *NANOG*, and *LIN28* sequences (Fig. 1a). In silico similarity analyses between the six reprogramming factors of the marmoset and the respective baboon sequences showed evolutionary conservation (> 96 %); only NANOG was less conserved (Table 1). Between days 25–30 after nucleofection, 10 to 15 primary colonies per plate (approx. 100 in total) with a morphology distinct from the non-reprogrammed fibroblasts were identified; new colonies appeared until day 60. Considering the number of colonies identified in this experiment, the overall reprogramming efficiency in this experiment is 0.0001 % (∼100 colonies$/1\times 10^{6}$ transfected cells during the course of the experiment – more colonies could have developed after termination of the reprogramming period) in relation to the total fibroblast transfected and 0.0063 % (12.5 colonies per primary plate/$0.2\times 10^{5}$ cells per primary plate) in relation to the puromycin resistant cells. Colonies had a stable morphology independent of the passage number and resembled other primate iPSCs in feeder culture. The colonies presented clear borders, and compact structure, and the cells had a high nuclei/cytoplasm ratio (Fig. 1a). During the first passages (approx. until passage 5) colonies with good morphology were picked manually to stabilize the different lines in culture. Then five colonies meeting the morphological PSC criteria were selected and further passaged and characterized (Fig. 1a). As a first screen for pluripotency, alkaline phosphatase activity was evaluated between passage 10 and 15. Generated iPSCs lines show alkaline phosphatase (AP) activity (Fig. 1b). Karyotyping was performed for biPSC1 and biPSC4. The cytogenetic analysis revealed a numerically normal karyotype of a female baboon without any evidence of structural chromosomal abnormalities (Fig. 1c).

**Table 1:** Similarity of the six factors used for reprogramming between marmoset and baboon.

	cDNA (%)	Protein (%)
SOX2	94.06	99.37
OCT4	85.37	96.03
KLF4	87.54	100
LIN28	96.98	98.56
c-MYC	82.84	97.72
NANOG	84.75	88.59

### 2.2 Detection of pluripotency markers

**Figure 2:**
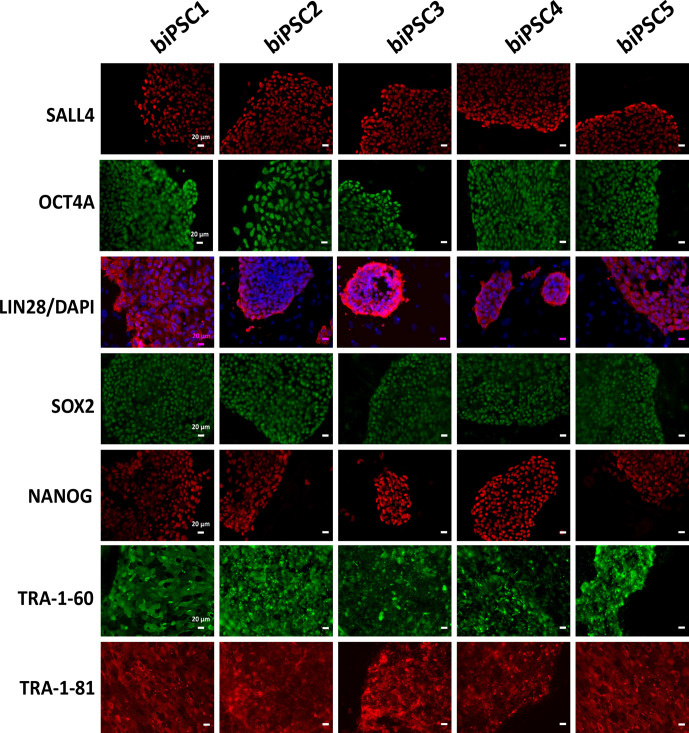
biPSC characterization by immunofluorescence staining. Detection of the pluripotency markers SALL4, OCT4A, LIN28, SOX2, NANOG, TRA-1-81, and TRA-1-60and. OCT4A, SOX2, NANOG, and LIN28 expression comes from both endogenous gene reactivation and exogenous transposon expression. SALL4, TRA-1-81, and TRA-1-60 result exclusively from endogenous expression as they are not contained in the reprogramming vector (scale bars 20 µm).

Each of the five lines generated expressed well-established pluripotency markers, including OCT4A (POU5F1), LIN28, TRA-1-60, TRA-1-81, SALL4, SOX2, and NANOG, as tested by immunostaining (Fig. 2). OCT4A, SALL4, NANOG, and SOX2 are nuclear, while LIN28 was detected in the cytoplasm. TRA1-60 and TRA-1-81 show membrane and cytoplasmic staining. These data indicate successful reprogramming (Fig. 2). Isotype controls were performed as a negative control (Fig. S1 in the Supplement).

**Figure 3:**
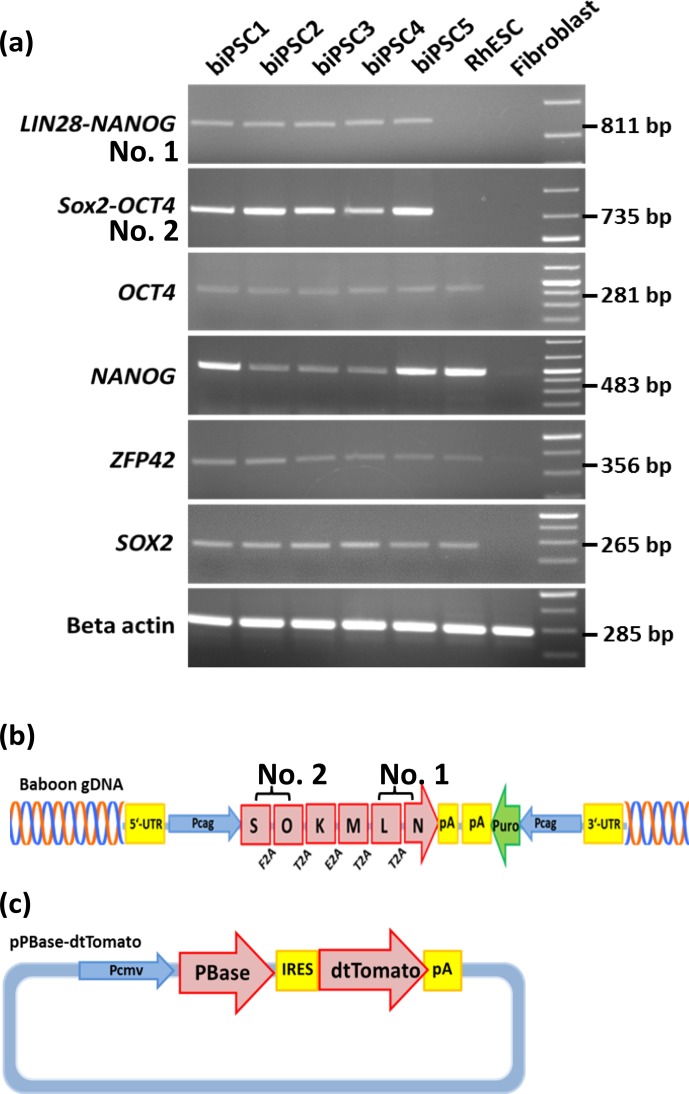
biPSC characterization by RT-PCR. **(a)** Expression analysis of the five baboon iPSCs lines (DPZ_biPSC1-5). In the absence of a baboon ESC line, rhesus embryonic stem cells (rhESC) were used as a positive control and baboon primary fibroblasts as a biological negative control (fibroblast). A 1 Kb Plus DNA Ladder was used as size marker. RT-PCR analysis of the iPSCs lines for the endogenous pluripotency factors *OCT4A*, *NANOG*, *ZFP42*, and *SOX2* as well as for the reprogramming cassette expression at two different sites, namely *SOX2*–*OCT4* (beginning of the transposon; no. 2 – see **b**), *LIN28*–*NANOG* (end of the transposon; no. 1). **(b)** *piggyBac* transposon used in this study, coding for the marmoset (*Callithrix jacchus*) reprogramming factors, *SOX2* (S), *OCT4* (O), *KLF4* (K), c-*MYC* (M), *LIN28* (L), and *NANOG *(N). 2A peptides substituting the stop codons were inserted between the reprogramming genes. The expression of the factors is driven by a CAG promoter. The puromycin resistance gene (Puro) is expressed under the control of an independent CAG promoter. **(c)** The *pBase-dtTomato* construct was transiently co-expressed with the vector shown in **(b)** and facilitates the integration of the transposon in the genome. Transposase expression is driven by the cytomegalovirus promoter (Pcmv).

We performed reverse transcription polymerase chain reaction (RT-PCR) to corroborate the expression of these factors at the messenger RNA (mRNA) level and to discriminate between endogenous expression and exogenous expression from the transposon (Fig. 3). To assess the expression of the transposon-derived transcript, primers were designed to demonstrate the presence of the 5${}^{\prime }$-end of the synthetic transposon transcript covering the fused *SOX2*–*OCT4* sequences, and at the 3${}^{\prime }$-end *LIN28*–*NANOG* (Fig. 3a and b). The transcript analysis was performed at passage 15 to 30. None of the lines showed the absence of the exogenous transcript according to the semiquantitative RT-PCR analysis. Although the *piggyBac* expression is still active, the biPSC lines present a comparable expression of endogenous *OCT4A, ZFP42*, and *SOX2* to rhesus monkey embryonic stem cells (rhESCs), which were used in the absence of a baboon ESC line as a positive control (Fig. 3). Only the intensity of the *NANOG* band was considerably weaker in some baboon iPSC lines than in the rhESC line.

### 2.3 Feeder-dependent and feeder-free culture of iPSCs

**Figure 4:**
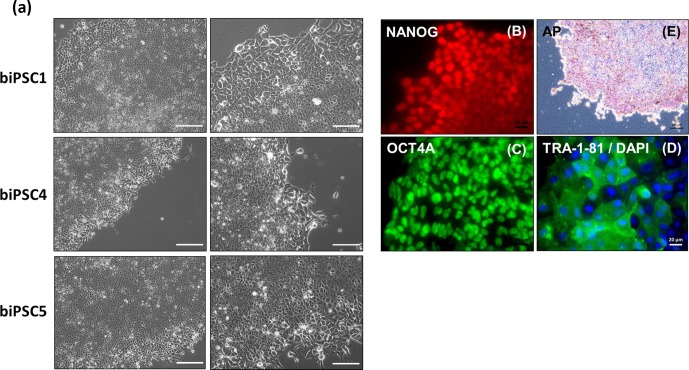
biPSCs adaptation to feeder-free conditions. **(a)** Bright field pictures of DPZ_biPSC1, DPZ_biPSC4, and DPZ_biPSC5 in culture on Geltrex with E8 medium (scale bar, **a** 200 µm; **b–d** 20 µm and **e** 100 µm). **(b–d)** DPZ_biPSC1 immunofluorescence staining, for **(b)** NANOG, **(c)** OCT4A, and **(d)** TRA1-81 to confirm that the lines remain undifferentiated after the adaptation to the feeder-free conditions (scale bar 20 µm). **(e)** Alkaline phosphatase staining of DPZ_biPSC1 under feeder-free conditions (scale bar represents 100 µm).

Three biPSC lines (DPZ_biPSC1, 4, and 5) were adapted to feeder-free conditions. The cells proliferated and remained undifferentiated in standard human iPSCs culture conditions, using Geltrex as an attachment matrix and Essential 8 medium (Fig. 4). The morphology of the colonies changed during the process of adaptation, and the lines stabilized after about three passages after the switch to the new culture conditions. Although adaptation of all five biPSC to feeder-free conditions was tested, only three of the lines were stable in the new culture conditions. The lines under feeder-free conditions maintained the typical cellular and colony morphology, i.e., small cells with a high nuclei/cytoplasm ratio. The colony morphology changed slightly in the new conditions; the rather regular borders seen on feeders changed to a more fringy appearance (Fig. 4a, also compare with Fig. 1a). After obtaining a homogenous undifferentiated morphology throughout the culture plate, it was possible to passage the cells by gentle dissociation using Versene solution instead of manual picking (Fig. 4a). Splitting using collagenase type IV and accutase was tested but led to a higher degree of morphological differentiation (data not shown). Five to ten passages after adaptation, a basic characterization was performed to check if the iPSCs remained undifferentiated. Expression of NANOG and OCT4A as pluripotency-specific markers as well as TRA-1-81 and AP indicated that the cells remained pluripotent even under feeder-free conditions (Fig. 4a–e).

### 2.4 Baboon iPSC differentiation by teratoma formation

**Figure 5:**
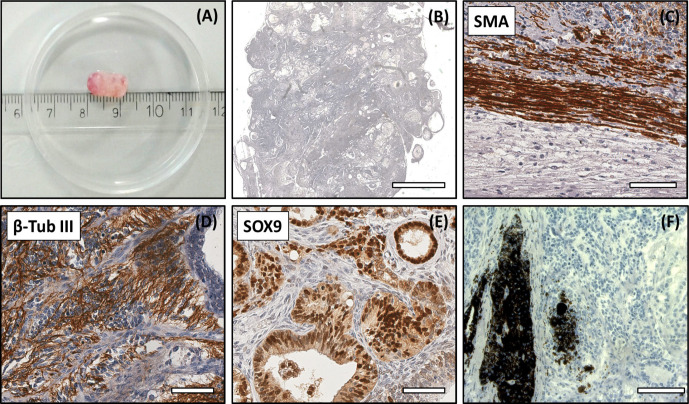
Analysis of the DPZ_biPSC 1 teratoma. Tumor tissue was immunohistochemically analyzed for the expression of representative markers of the three germ layers: SOX9, β-tubulin 3, and smooth muscle actin (SMA). **(a)** Macroscopic view of DPZ_biPSC 1 teratoma. **(b)** Overview of a H&E-stained teratoma section showing the structural heterogeneity (bar represents 2 mm). **(c)** Smooth muscle actin (SMA) staining indicating mesodermal differentiation. **(d)** β-Tubulin 3 staining (β-Tub III) indicating ectodermal differentiation. **(e)** SOX9 staining. SOX9 is a marker of endodermal progenitor and stem cells. **(f)** Pigmented cells (no immunohistochemical staining) indicating ectodermal differentiation (bars in **c–f** represent 70 µm).

To demonstrate the differentiation potential of the generated iPSCs functionally, the teratoma formation assay was exemplarily performed for the DPZ_biPSC lines 1 and 5 by injecting $∼1\times 10^{6}$ iPSCs subcutaneously into immunodeficient mice. DPZ_biPSC1 was injected after adaptation to feeder-free conditions, in contrast to DPZ_biPSC5, which remained under feeder-dependent conditions until injection. Tumors developed within 15 weeks. According to the type of culture (with MEFs or feeder-free on Geltrex), the lines were injected together with MEFs (DPZ_biPSC5) or Geltrex (DPZ_biPSC1) as an initial support for survival after injection (Fig. 5).

Both lines formed tumors that were analyzed when their diameter reached approximately 1 cm (Figs. 5a and S2a). The expression of markers of each embryonic germ layer was immunohistochemically analyzed. In a DPZ_biPSC1-derived tumor SOX9 (primitive endodermal epithelium), β-tubulin 3 (ectoderm), and smooth muscle actin (SMA) (mesoderm) were detected, demonstrating the pluripotency of this line (Fig. 5c–e). Also, clusters of pigmented cells were detected (Fig. 5f), which also indicate ectoderm. The DPZ_biPSC5-derived teratoma similarly showed differentiated cells expressing the respective markers but also contained clusters that remained undifferentiated as indicated by OCT4A- (Fig. S2c) and NANOG-positive (not shown) cells, even 15 weeks after injection.

## 3 Methods

### 3.1 Animals and animal housing; ethics statement

The German Primate Center is registered and authorized by the local and regional veterinary governmental authorities (reference number: 122910.3311900, PK Landkreis, Göttingen). The DPZ runs self-sustaining colonies of different primate species including baboons. The animal from which the cells were obtained was sacrificed at the age of 12 years in the context of an unrelated approved study. Skin samples were made available during necropsy to the Platform Degenerative Diseases of the DPZ.

### 3.2 Cell culture

#### 3.2.1 Isolation of baboon primary fibroblasts

Skin tissue pieces of approximately 1×1 cm were dissected to remove subcutaneous adipose tissue and washed three times with phosphate-buffered saline (PBS) buffer with antibiotics (1 %, v/v, penicillin/streptomycin, Gibco, 0.25 µg mL${}^{-1}$ amphotericin B, Sigma). The skin was minced with scalpels and digested in Dulbecco's Modified Eagle Medium (DMEM) supplemented with 10 mg mL${}^{-1}$ collagenase type IV (Gibco) for 3 h at 37 ${}^{\circ }$C at a constant rotation at 800 rpm. After the digestion, the remaining non-digested skin fragments were removed, and the cell suspension was centrifuged (300 g, 5 min, RT). The supernatant was discarded, and the pellet resuspended in Rh15 medium (DMEM, Gibco, 15 %, v/v; fetal bovine serum, Gibco, 1 %, v/v; penicillin/streptomycin, Gibco, 0.25 µg mL${}^{-1}$; amphotericin B, Sigma, 1 %, v/v; Minimum Essential Medium (MEM) non-essential amino acids solution, Gibco; 2 mM GlutaMAX, Gibco) and plated onto two 10 cm diameter gelatine-coated culture dishes (0.1 % gelatine; Sigma). Fibroblasts were passaged using StemPro accutase (Thermo Fisher).

#### 3.2.2 Mouse embryonic fibroblasts (MEFs)

Gamma-irradiated MEFs were used as feeder cells. Their generation and culture were described previously (Debowski et al., 2015).

#### 3.2.3 Nucleofection

For reprogramming the *piggyBac* NHP six-factor transposon system was used (Debowski et al., 2015). Baboon fibroblasts ($1\times 10^{6}$ cells) were nucleofected (6 µg pDNA) after at least three passages in culture using the 4-D Nucleofector (Lonza), with P2 Primary Cell 4D-Nucleofector^®^ X Kit L (Lonza; program CA-137). A pmax-GFP (green fluorescent protein) vector was used as a positive control for nucleofection.

#### 3.2.4 Reprogramming procedure

After nucleofection, the efficiency was estimated by the expression of the reporters in the *pBase-dtTomato* transposase (Fig. 3c) and the pmax-GFP. Antibiotic selection was started 2 d after nucleofection by supplementation of the Rh15 medium for 5 d with 1.5 µg mL${}^{-1}$ puromycin (Sigma). After selection, the cells were transferred at day 6 after nucleofection onto gelatine-coated 10 cm plates with MEFs ($0.2\times 10^{5}$ cells per plate) and cultured in embryonic stem cell medium (ESM) (KO-DMEM, Gibco; 20 %, v/v, KnockOut Serum Replacement, Gibco;, 1 %, v/v, penicillin/streptomycin, Gibco; 0.25 µg mL${}^{-1}$ amphotericin B, Sigma; 1 %, v/v, MEM nonessential amino acid solution, Gibco; 2 mM GlutaMAX, Gibco; 50 µM 2-mercaptoethanol, Gibco) supplemented with 10 ng mL${}^{-1}$ fibroblast growth factor (FGF, ThermoFisher). During the first 6 d of culture on MEFs, ESM was supplement with 2 mM valproic acid (Calbiochem). The first colonies appeared at day 25, and new colonies were picked until day 60. Colonies were picked manually from the primary plate to fresh plates with MEFs. Around passage 5, colonies were stable enough to stop manual picking and passaged using 1 mg mL${}^{-1}$ collagenase type IV (Gibco).

Freezing of the cells was performed using ESC medium with 20 %DMSO (dimethylsulfoxid, DMSO; Sigma at -150 ${}^{\circ }$C).

### 3.3 RT-PCR to evaluate the expression of endogenous and exogenous (*piggyBac*) pluripotency factors

RNA extraction was performed from frozen cell pellets using the RNAeasy Mini Kit (Qiagen). For complementary DNA (cDNA) synthesis, the omniscript RT kit (Qiagen) was used according to the manufacturer's protocol using 1 µg mRNA per reaction. Residual genomic DNA (gDNA) was removed using the RNase-Free DNase kit (Qiagen). Oligonucleotides (Sigma) are detailed in Table 2. Primers to analyze endogenous expression were designed specifically for the 3${}^{\prime }$UTR of each pluripotency gene, which is not included in the reprogramming cassette. Specific amplification of exogenous transcripts was performed by designing primers located in two consecutive coding sequences of the *piggyBac* not present in the wild type genome. In the absence of baboon ESCs, rhesus monkey embryonic stem cells (rhESC; line 366.4) (Thomson et al., 1995) were used as a positive control and baboon fibroblasts as a negative control. Beta-actin was used as a housekeeping gene for normalization.

**Table 2:** Oligonucleotides (Sigma) used for the cDNA amplification of the endogenous and exogenous pluripotency factors and *ACTB* used as housekeeping gene.

Name	Primer sequence 5${}^{\prime }$3${}^{\prime }$
SOX2-OCT4_fw	TCTTCCTCGCACTCCAGGGC
SOX2-OCT4_rev	CAGGGTGATCCTCTTCTGCTTC
LIN28-NANOG_fw	AGCCATATGGTAGCCTCATGTCC
LIN28-NANOG_rev	GGTTGCTCCAGGTTGAATTGC
OCT4_fw	GAAGGATGTGGTCCGAGTGT
OCT4_rev	AGGCGCCTCAGTTTGAATGC
NANOG_fw	GCAATGGTGTGACTCAGAAGG
NANOG_rev	TTCAGCCAGTGCCCAGACTG
ZFP42_fw	CAGAGCCCATTCTTGGTCGG
ZFP42_rev	CATTTCCGCCTGCAGGTCTT
SOX2_fw	GGCATGGCTCTTGGTTCCAT
SOX2_rev	TGTCCTGCCCTCACATGTGC
ACTB _fw	GACCTGACTGACTACCTCATG
ACTB_rev	GGTAGTTTCGTGGATGCCACA

### 3.4 Alkaline phosphatase

Alkaline phosphatase activity was demonstrated using the leukocyte alkaline phosphatase kit (Sigma) according to the manufacturer's instructions.

### 3.5 Immunofluorescence staining

Cells were cultured on coverslips until the colonies reach 60 %–80 % confluence. For fixation, the plates were washed three times with PBS and fixed using 4 % paraformaldehyde (PFA) (v/v) for 20 min at room temperature. Before blocking, cells were washed three more times with PBS. Blocking was performed using 1 % bovine serum albumin (BSA) in PBS for surface markers or 1 % BSA in PBS supplemented with TritonX-100 (0.1 %, Sigma) for permeabilization in the case of staining intracellular markers. The primary antibody was diluted in 1 % BSA in PBS and the cells were incubated for 1 h at 37 ${}^{\circ }$C. The washing step described above was repeated between first and second antibody incubations. Alexa488-coupled secondary antibodies (Life Technologies) diluted in 1 % BSA in PBS were also applied for 1 h at 37 ${}^{\circ }$C. Cells were finally incubated in 4${}^{\prime }$,6-diamidino-2-phenylindole (DAPI) diluted in PBS (05 µg mL${}^{-1}$; Roth) for 10 min to stain the nuclei. The coverslips were mechanically detached and mounted using citifluor mountant medium (CITIFLUOR ltd.). Immunofluorescence images were taken with a Zeiss Observer Z1 (Zeiss). The primary antibodies used and their dilutions were OCT4A (cell signalling OCT-4A C52G3, 1:1600), SOX2 (cell signalling C70B1, 1:200), NANOG (cell signalling D73G4, 1:400), TRA 1-60 (eBioscience 14-8863, 1:100), TRA 1-81 (eBioscience 14-8883, 1:100), LIN28 (cell signalling A177, 1:100), and SALL4 (Abcam ab57577, 1:200). As secondary antibodies, Alexa-488 conjugated donkey anti-mouse IgG (H+L) (Life technologies A21202, 1:1000), donkey anti-rabbit IgG (H+L) (Invitrogen A-21206, 1:1000), and goat anti-mouse immunglobulin M (IgM) (H+L) (Invitrogen A-21042, 1:1000) were used.

### 3.6 Teratoma formation and histological analysis

For the teratoma assay, cells were prepared for injection by mixing $8\times 10^{5}$ biPSC with $2\times 10^{5}$ MEFs in the case of injection with feeders and $1\times 10^{6}$ biPSCs in the case of feeder-free lines. Cells were diluted in PBS substituted with Geltrex solution (0.1 mg mL${}^{-1}$, ThermoFisher) with a final volume of 120 µL per injection. Immunodeficient RAG2${}^{-/-}\gamma$c ${}^{-/-}$mice were injected subcutaneously with the different cell lines. The mice were checked regularly, and the formation of tumors was monitored by palpation. Teratomas were isolated when reaching a diameter of 1 cm around 15 weeks after the injection. The immunohistochemical staining protocol has been described previously (Eildermann et al., 2012). The antibodies used for immunohistochemical staining were anti-β-tubulin III (Sigma, T8660, 1:600), anti-SMA (Sigma, A2547, 1:1000), anti-SOX9 (Millipore, AB5535, 1:1000), and anti-OCT4A (cell signalling, 2890, 1:1000).

### 3.7 Adaptation to feeder-free conditions

Baboon iPSC colonies were manually dissociated and transferred using a pipet to a 7 cm culture dish coated with Geltrex (Thermo Fisher Scientific). Cells were cultured in Essential 8 medium (E8; Thermo Fisher). For the first 2 d after the transfer, the medium was supplemented with ROCK inhibitor pro-survival compound (PSC DDD00033325 – Calbiochem; 5 µM). Colonies were initially manually picked and then split with Versene solution (Thermo Fisher). Dilution factors during passaging of the cells varied between 1:10 and 1:20. Freezing of the cells under feeder-free conditions was done using the Essential 8 medium supplemented with 20 % DMSO (Sigma) and 10 µM PSC.

### 3.8 Karyotyping

Baboon iPSCs were treated with demecolcine solution (380 ng mL${}^{-1}$; Sigma-Aldrich) for 5 h at 37 ${}^{\circ }$C. Cells were detached with accutase (Thermo Fisher) for 1 min at 37 ${}^{\circ }$C collected in a 15 mL tube and centrifuged at 200g, 5 min (RT). The supernatant was discarded, and the cell pellet resuspended in 1 mL ESM by tapping the tube carefully. Pre-warmed (37 ${}^{\circ }$C) hypotonic KCl/sodium citrate solution (1/1, 0.075 M/3.8 mM) was added dropwise to the cell suspension while shaking the tube carefully (approx. 8 mL). After 15 min of incubation at 37 ${}^{\circ }$C, the cells were centrifuged (1000 rpm, 8 min (RT)) and the supernatant discarded. Pre-cooled (-20 ${}^{\circ }$C) fixative (3:1 methanol/glacial acetic acid v/v) was then added dropwise to the cell suspension while shaking the tube carefully. The cells were incubated on ice for 10 min and then centrifuged (fixation and centrifugation steps were repeated three times). Fixed cells were dropped onto slides and baked at 60 ${}^{\circ }$C overnight. Dry samples were immersed in 0.15 % trypsin/NaCl (Biochrom) for 50–60 s and stained with 5 % Giemsa (Merck). A total of 11 G-banded metaphases were analyzed from the selected cell lines. Karyotypes of baboon iPSCs were arranged according to Moore et al. (1999) using the IKAROS imaging system (Metasystems).

### 3.9 Sequence comparison

The following sequences were used for the determination of the similarity between the marmoset and the baboon cDNA and protein, respectively: SOX2 (ENSCJAG00000008401/ENSPANG00000025489), OCT4A (ENSCJAG00000019789/ENSPANG00000007627), KLF4 (ENSCJAG00000016955/XM_003911399.2 and ENSPANG00000025718), LIN28 (ENSCJAG00000009796/ENSPANG00000009841), c-MYC (ENSCJAG00000012620/ENSPANG00000010418), and NANOG (ENSCJAG00000018999/ENSPANG00000009019).

## 4 Discussion

NHP iPSCs are of relevance for the preclinical evaluation of regenerative therapies (Phillips et al., 2014). This may particularly involve therapies of the central and peripheral nervous system, the eye, the heart, and the vascular system (Stevens et al., 2009; Grow et al., 2016a; Agrba et al., 2016; Längin et al., 2018; Shiba et al., 2016; Emborg et al., 2013; Wang et al., 2015; Chong and Murry, 2014). Marmosets and macaques (rhesus and cynomolgus monkeys) are the most frequently used NHP species in biomedical research, but they differ in some characteristics from humans, including body size (Behr, 2015). In terms of body size, immunological characteristics, and other features, the baboon can be an excellent alternative model to mimic human pathology. The similar anatomy, specifically organ size, and immunology of baboons are reflected in their crucial role in (xeno-) transplantation research (Bailey, 2009; Längin et al., 2018). Since different iPSC lines from the same species, even from the same animal, have different properties and may respond differently to external cues, it is expedient to have a representative number of baboon iPSC lines available (Ohi et al., 2011; Carcamo-Orive et al., 2017). So far, there are only very few baboon iPSC lines, and it has been reported that the reprogramming of cells particularly from this species is challenging in comparison to humans, macaques, or mice (Navara et al., 2013, 2018). Here, we report the generation and initial characterization of five new baboon iPSC lines and developed a robust protocol that can be used to easily increase the number of available lines.

We demonstrate that feeder-free culture of the novel baboon iPSC lines is possible, at least after initial derivation of the lines on MEFs. Although we tried to adapt all lines to feeder-free culture, this was not achieved for biPSC2 and 3. We believe that further fine-tuning of the feeder-free culture conditions is necessary to generate a robust protocol that works for all lines. Feeder-free culture is important concerning preclinical testing, upscaling, and the molecular analysis of pure cell populations which are not intermingled by xeno-genic cells. Furthermore, the feeder-free protocol may pave the way to fully xeno-free generation of NHP iPSC lines (Villa-Diaz et al., 2013; Nishizawa et al., 2014; Hakala et al., 2009). Finally, in the context of the three Rs (replace, reduce, refine) of animal research, a reduction in the number of animals used needs to be considered since MEFs are prepared from E12.5 mouse embryos.

In the present study, we successfully generated iPSC lines from an adult female baboon. A previous report on baboon iPSCs used embryonic fibroblasts, which are much more easily reprogrammable than adult fibroblasts according to our own unpublished and previously published data (Debowski et al., 2015; Imamura et al., 2012). Generally, we have shown that our approach using the *piggyBac* system is robust and reproducible. Furthermore, we speculate that the efficiency of reprogramming of neonatal or even embryonic fibroblasts, using the same protocol, will be higher than using adult fibroblasts. However, this was not possible to test in our study due to the lack of tissue samples from young or even prenatal baboons.

The *piggyBac* cassette can be removed from the cells without leaving a footprint in the genome. This has been shown in previous reports with human and mouse iPSCs (Malik and Mahendra, 2013; Patel and Yang, 2010; Woltjen et al., 2011; Yusa et al., 2009; Mohseni et al., 2009) and could also be done with the Baboon lines. The option to remove the reprogramming cassette is a clear advantage of this system in comparison to retroviral vectors, which are stably integrated into the genome (Navara et al., 2013). Moreover, even if retroviral vectors are flanked by recombination sites, e.g., loxP sites, facilitating their excision, the excision site remains mutated due to incomplete removal of the recombination site. Currently, we cannot exclude that pluripotency factor expression from the transposon also supports feeder-free culture. After future removal of the transposon, the continuous culture on Geltrex needs to be reevaluated.

Protein expression analysis of the pluripotency factors in our biPSCs lines shows that they express OCT4A, LIN28, NANOG, SOX2 and SALL4, and tumor rejection antigens (Fig. 2). However, transcript analysis by RT-PCR, which can – in contrast to antibodies – discriminate between endogenous and exogenous factors, showed two things: first, the endogenous genes encoding the pluripotency factors are all reactivated, though at different levels (Fig. 3). Secondly, the *piggyBac*-derived transcripts are still detectable and therefore will contribute to the signals detected on the protein level by immunostaining (Fig. 2). In parallel studies with marmoset monkey iPSCs generated using the same system, we observed a silencing of the transposon and that the contribution of the exogenous expression to the overall pluripotency factor expression was (at least on the transcript level) most likely lower than the expression from the endogenous reactivation of pluripotency genes (Debowski et al., 2015). We speculate that passage-number-dependent promoter methylation may influence the expression level of the *piggyBac* cassette. In fact, we have evidence from *piggyBac*-mediated reprogramming-derived rhesus iPSC lines that methylation of the CAG promoter driving the reprogramming cassette occurs (unpublished data). Although the exogenous transposon-encoded factors are still active in cultured cells, the teratoma assays show that the iPSC lines are not arrested in pluripotency by the forced expression of the reprogramming factors. In fact, the teratomas show that expression from the *piggyBac* cassette is strongly downregulated, or even switched off, since in almost all cells of the teratoma (except the few clusters of cells like the one shown in Fig. S2) we were not able to detect OCT4A and other cassette-encoded factors on the protein level by robust and sensitive immunohistochemistry (Aeckerle et al., 2015; Wolff et al., 2019).

Altogether, as demonstrated by five novel iPSCs lines from an adult female olive baboon, we have established a robust protocol for the generation of adult baboon iPSC lines. In general, the fully reversible six-factor *piggyBac* system with monkey reprogramming factors seems to be an efficient tool for the generation of NHP iPSCs in general since we have successfully used the *piggyBac* system also for the generation of difficult-to-reprogram marmoset (Debowski et al. 2015) as well as of rhesus cells (unpublished). In cases where non-integrating approaches like the Sendai virus method do not work reliably, the *piggyBac* system represents an efficient alternative.

## Supplement

10.5194/pb-6-75-2019-supplement The supplement related to this article is available online at: https://doi.org/10.5194/pb-6-75-2019-supplement.

## Data Availability

Data sharing is generally not applicable to this article as this study analyses mostly qualitative data and no large datasets were generated during this study. Original data are available upon request.

## References

[bib1.bib1] Aeckerle N, Drummer C, Debowski K, Viebahn C, Behr R (2015). Primordial germ cell development in the marmoset monkey as revealed by pluripotency factor expression: suggestion of a novel model of embryonic germ cell translocation. Mol Hum Reprod.

[bib1.bib2] Agrba VZ, Porkhanov VA, Karal-Ogly DD, Leontyuk AV, Kovalenko AL, Sholin IY, Gvozdik TE, Ignatova IE, Agumava AA, Chuguev YP, Gvaramiya IA, Lapin BA (2016). Transplantation of Simian Mesenchymal Stem Cells to Baboons with Experimentally Induced Myocardial Infarction. B Exp Biol Med.

[bib1.bib3] Bailey L, Williams-Blangero S, Tardif SD, VandeBerg JL (2009). The Baboon in Biomedical Research – The Baboon in Xenotransplant Research.

[bib1.bib4] Behr R., Weinbauer GF, Vogel F (2015). Primate biologics research at a crossroads, Potential of Genetically
Modified Nonhuman Primate Models for Biomedicine.

[bib1.bib5] Carcamo-Orive I, Hoffman GE, Cundiff P, Beckmann ND, D'Souza SL, Knowles JW, Patel A, Papatsenko D, Abbasi F, Reaven GM, Whalen S, Lee P, Shahbazi M, Henrion MYR, Zhu K, Wang S, Roussos P, Schadt EE, Pandey G, Chang R, Quertermous T, Lemischka I (2017). Analysis of Transcriptional Variability in a Large Human iPSC Library Reveals Genetic and Non-genetic Determinants of Heterogeneity. Cell Stem Cell.

[bib1.bib6] Chong JJH, Murry CE (2014). Cardiac Regeneration Using Pluripotent Stem Cells – Progression to Large Animal Models. Stem Cell Res.

[bib1.bib7] Cox LA, Comuzzie AG, Havill LM, Karere GM, Spradling KD, Mahaney MC, Nathanielsz PW, Nicolella DP, Shade RE, Voruganti S, VandeBerg JL (2013). Baboons as a model to study genetics and epigenetics of human disease. ILAR J.

[bib1.bib8] Debowski K, Warthemann R, Lentes J, Salinas-Riester G, Dressel R, Langenstroth D, Gromoll J, Sasaki E, Behr R (2015). Non-viral generation of marmoset monkey iPS cells by a six-factor-in-one-vector approach. PLoS One.

[bib1.bib9] Didier ES, MacLean AG, Mohan M, Didier PJ, Lackner AA, Kuroda MJ (2016). Contributions of Nonhuman Primates to Research on Aging. Vet Pathol.

[bib1.bib10] Eildermann K, Aeckerle N, Debowski K, Godmann M, Christiansen H, Heistermann M, Schweyer S, Bergmann M, Kliesch S, Gromoll J, Ehmcke J, Schlatt S, Behr R (2012). Developmental expression of the pluripotency factor sal-like protein 4 in the monkey, human and mouse testis: Restriction to premeiotic germ cells. Cells Tissues Organs.

[bib1.bib11] Emborg ME, Liu Y, Xi J, Zhang X, Yin Y, Lu J, Joers V, Swanson C, Holden JE, Zhang S (2013). Induced Pluripotent Stem Cell-Derived Neural Cells Survive and Mature in the Nonhuman Primate Brain. Cell Rep.

[bib1.bib12] Grow DA, McCarrey JR, Navara CS (2016). Advantages of nonhuman primates as preclinical models for evaluating stem cell-based therapies for Parkinson's disease. Stem Cell Res.

[bib1.bib13] Grow DA, Simmons DV, Gomez JA, Wanat MJ, McCarrey JR, Paladini CA, Navara CS (2016). Differentiation and Characterization of Dopaminergic Neurons From Baboon Induced Pluripotent Stem Cells. Stem Cells Transl Med.

[bib1.bib14] Hakala H, Rajala K, Ojala M, Panula S, Areva S, Kellomäki M, Suuronen R, Skottman H (2009). Comparison of Biomaterials and Extracellular Matrices as a Culture Platform for Multiple, Independently Derived Human Embryonic Stem Cell Lines. Tissue Eng.

[bib1.bib15] Imamura M, Okuno H, Tomioka I, Kawamura Y, Lin ZY-C, Nakajima R, Akamatsu W, Okano HJ, Matsuzaki Y, Sasaki E, Okano H (2012). Derivation of Induced Pluripotent Stem Cells by Retroviral Gene Transduction in Mammalian Species. Methods Mol Biol.

[bib1.bib16] Kim K-Y, Hysolli E, Park I-H (2012). Reprogramming Human Somatic Cells into Induced Pluripotent Stem Cells (iPSCs) Using Retroviral Vector with GFP. J Vis Exp.

[bib1.bib17] Kimbrel EA, Lanza R (2015). Current status of pluripotent stem cells: Moving the first therapies to the clinic. Nat Rev Drug Discov.

[bib1.bib18] Kobayashi Y, Okada Y, Itakura G, Iwai H, Nishimura S, Yasuda A, Nori S, Hikishima K, Konomi T, Fujiyoshi K, Tsuji O, Toyama Y, Yamanaka S, Nakamura M, Okano H (2012). Pre-Evaluated Safe Human iPSC-Derived Neural Stem Cells Promote Functional Recovery after Spinal Cord Injury in Common Marmoset without Tumorigenicity. PLoS One.

[bib1.bib19] Längin M, Mayr T, Reichart B, Michel S, Buchholz S, Guethoff S, Dashkevich A, Baehr A, Egerer S, Bauer A, Mihalj M, Panelli A, Issl L, Ying J., Fresch AK, Buttgereit I, Mokelke M, Radan J, Werner F, Lutzmann I, Steen S, Sjöberg T, Paskevicius A, Qiuming L, Sfriso R, Rieben R, Dahlhoff M, Kessler B, Kemter E, Klett K, Hinkel R, Kupatt C, Falkenau A, Reu S, Ellgass R, Herzog R, Binder U, Wich G, Skerra A, Ayares D, Kind A, Schönmann U, Kaup F-J, Hagl C, Wolf E, Klymiuk N, Brenner P, Abicht J-M (2018). Consistent success in life-supporting porcine cardiac xenotransplantation. Nature.

[bib1.bib20] Malik N, Mahendra SR (2013). A Review of the Methods for Human iPSC Derivation. Methods Mol Biol.

[bib1.bib21] Mohseni P, Woltjen K, Kaji K, Paca A, Mileikovsky M, Norrby K (2009). Virus-free induction of pluripotency and subsequent excision of reprogramming factors. Nature.

[bib1.bib22] Moore CM, Janish C, Eddy CA, Hubbard GB, Leland MM, Rogers J (1999). Cytogenetic and Fertility Studies of a Rheboon, Rhesus Macaque (Macaca mulatta) Baboon (Papio hamadryas) Cross: Further Support for a Single Karyotype Nomenclature. Am J Phys Anthropol.

[bib1.bib23] Navara CS, Hornecker J, Grow D, Chaudhari S, Hornsby PJ, Ichida JK, Eggan K, McCarrey JR (2013). Derivation of induced pluripotent stem cells from the baboon: a nonhuman primate model for preclinical testing of stem cell therapies. Cell Reprogram.

[bib1.bib24] Navara CS, Chaudhari S, McCarrey JR (2018). Optimization of culture conditions for the derivation and propagation of baboon (Papio anubis) induced pluripotent stem cells. PLoS One.

[bib1.bib25] Nishizawa M, Yamanaka S, Ichisaka T, Takizawa N, Taniguchi Y, Toyoda T, Osafune K, Takahashi J, Sekiguchi K, Nakagawa M, Doi D, Senda S, Asano K, Yoshida Y, Morizane A (2014). A novel efficient feeder-free culture system for the derivation of human induced pluripotent stem cells. Sci Rep.

[bib1.bib26] Nishizawa M, Chonabayashi K, Nomura M, Tanaka A, Nakamura M, Inagaki A, Nishikawa M, Takei I, Oishi A, Tanabe K, Ohnuki M, Yokota H, Koyanagi-Aoi M, Okita K, Watanabe A, Takaori-Kondo A, Yamanaka S, Yoshida Y (2016). Epigenetic Variation between Human Induced Pluripotent Stem Cell Lines Is an Indicator of Differentiation Capacity. Cell Stem Cell.

[bib1.bib27] Ohi Y, Qin H, Hong C, Blouin L, Polo JM, Guo T, Qi Z, Downey SL, Manos PD, Rossi DJ, Yu J, Hebrok M, Hochedlinger K, Costello JF, Song JS (2011). Incomplete DNA methylation underlies a transcriptional memory of the somatic cell in human iPS cells. Nat Cell Biol.

[bib1.bib28] Patel M, Yang S (2010). Advances in Reprogramming Somatic Cells to Induced Pluripotent Stem Cells. Stem Cell Rev.

[bib1.bib29] Phillips KA, Bales KL, Capitanio JP, Conley A, Czoty PW, 't Hart BA, Hopkins WD, Hu SL, Miller LA, Nader MA, Nathanielsz PW, Rogers J, Shively CA, Voytko ML (2014). Why primate models matter. Am J Primatol.

[bib1.bib30] Rogers J, Hixson JE (1997). Baboons as an Animal Model for Genetic Studies of Common Human Disease. Am J Hum Genet.

[bib1.bib31] Shearer MH, Dark RD, Chodosh J, Kennedy RC (1999). Comparison and Characterization of Immunoglobulin G Subclasses among Primate Species Comparison and Characterization of Immunoglobulin G Subclasses among Primate Species. Clin Diagn Lab Immun.

[bib1.bib32] Shiba Y, Gomibuchi T, Seto T, Wada Y, Ichimura H, Tanaka Y, Ogasawara T, Okada K, Shiba N, Sakamoto K, Ido D, Shiina T, Ohkura M, Nakai J, Uno N, Kazuki Y, Oshimura M, Minami I, Ikeda U (2016). Allogeneic transplantation of iPS cell-derived cardiomyocytes regenerates primate hearts. Nature.

[bib1.bib33] Simerly CR, Navara C, Castro CA, Turpin JC, Redinger CJ, Mich-Basso JD, Jacoby ES, Jacoby ES, Grund KJ, McFarland DA, Oliver SL, Ben-Yehudah A, Carlisle DL, Frost P, Penedo C, Hewitson L, Schatten G (2009). Establishment and characterization of baboon embryonic stem cell lines:
An Old World Primate model for regeneration and transplantation research. Stem Cell Res.

[bib1.bib34] Sosa E, Kim R, Rojas EJ, Hosohama L, Hennebold JD, Orwig KE, Clark AT (2017). An integration-free, virus-free rhesus macaque induced pluripotent stem cell line (riPSC89) from embryonic fibroblasts. Stem Cell Res.

[bib1.bib35] Stevens KR, Bendixen K, Regnier M, Dupras SK, Muskheli V, Kreutziger KL, Reinecke H, Nourse MB, Korte FS, Murry CE (2009). Physiological function and transplantation of scaffold-free and vascularized human cardiac muscle tissue. P Natl Acad Sci USA.

[bib1.bib36] Takahashi K, Yamanaka S (2006). Induction of Pluripotent Stem Cells from Mouse Embryonic and Adult Fibroblast Cultures by Defined Factors. Cell.

[bib1.bib37] Takahashi K, Tanabe K, Ohnuki M, Narita M, Ichisaka T, Tomoda K (2007). Induction of Pluripotent Stem Cells from Adult Human Fibroblasts by Defined Factors. Cell.

[bib1.bib38] Thomson JA, Kalishman J, Golos TG, Durning M, Harris CP, Becker RA, Hearn JP (1995). Isolation of a primate embryonic stem cell line. P Natl Acad Sci USA.

[bib1.bib39] Tiburcy M, Hudson JE, Balfanz P, Schlick S, Meyer T, Liao MLC, Levent E, Raad F, Zeidler S, Wingender E, Riegler J, Wang M, Gold JD, Kehat I, Wettwer E, Ravens U, Dierickx P, Van Laake LW, Goumans MJ, Khadjeh S, Toischer K, Hasenfuss G, Couture LA, Unger A, Linke WA, Araki T, Neel B, Keller G, Gepstein L, Wu JC, Zimmermann WH (2017). Defined engineered human myocardium with advanced maturation for applications in heart failure modeling and repair. Circulation.

[bib1.bib40] Turnbull IC, Karakikes I, Serrao GW, Backeris P, Lee JJ, Xie C, Senyei G, Gordon RE, Li RA, Akar FG, Hajjar RJ, Hulot JS, Costa KD (2014). Advancing functional engineered cardiac tissues toward a preclinical model of human myocardium. FASEB J.

[bib1.bib41] Varilly P, Chandler D (2008). An age-old paradigm challenged: Old baboons generate vigorous humoral immune responses to LcrV, a plague antigen. J Immunol.

[bib1.bib42] Villa-Diaz L.G, Ross AM, Lahann J, Krebsbach PH (2013). Concise review: The evolution of human pluripotent stem cell culture: From feeder cells to synthetic coatings. Stem Cells.

[bib1.bib43] Wang S, Zou C, Fu L, Wang B, An J, Song G, Wu J, Tang X, Li M, Zhang J, Yue F, Zheng C, Chan P, Zhang YA, Chen Z (2015). Autologous iPSC-derived dopamine neuron transplantation in a nonhuman primate Parkinson's disease model. Cell Discov.

[bib1.bib44] Wolff E, Suplicki MM, Behr R (2019). Primordial germ cells do not migrate along nerve fibres in marmoset monkey and mouse embryos. Reproduction.

[bib1.bib45] Woltjen K, Hämäläinen R, Kibschull M, Mileikovsky M, Nagy A (2011). Transgene-free production of pluripotent stem cells using piggyBac transposons. Methods Mol Biol.

[bib1.bib46] Yamanaka S, Okita K, Sato Y, Saji H, Okamoto S, Takahashi M, Tanabe K, Takahashi J, Tezuka K, Shibata T, Hong H, Matsumura Y, Kunisada T, Nakagawa M, Morizane A, Okada A (2011). A more efficient method to generate integration-free human iPS cells. Nat Methods.

[bib1.bib47] Yu J, Hu K, Smuga-otto K, Tian S, Stewart R, Igor I, Thomson JA (2009). Human Induced Pluripotent Stem Cells Free of Vector and Transgene Sequences. Science.

[bib1.bib48] Yusa K, Rad R, Takeda J, Bradley A (2009). Generation of transgene-free induced pluripotent mouse stem cells by the piggyBac transposon. Nat Methods.

[bib1.bib49] Zhang X, Cao H, Bai S, Huo W, Ma Y (2017). Differentiation and characterization of rhesus monkey atrial and ventricular cardiomyocytes from induced pluripotent stem cells. Stem Cell Res.

