# Supplementary material for: Baboon induced pluripotent stem cell generation by piggyBac transposition of reprogramming factors
Source: Primate Biol. 2019 Jul 29;6(2):75–86. doi: 10.5194/pb-6-75-2019 (PMC7041535; doi:10.5194/pb-6-75-2019)
Supplement: The supplement related to this article is available online at: https://doi.org/10.5194/pb-6-75-2019-supplement. [file pb-6-75-supplement.pdf]

Supplement of Primate Biol., 6, 75–86, 2019
https://doi.org/10.5194/pb-6-75-2019-supplement
© Author(s) 2019. This work is distributed under
the Creative Commons Attribution 4.0 License.

Supplement of

Baboon induced pluripotent stem cell generation by *piggyBac* transposition of reprogramming factors

Ignacio Rodriguez-Polo et al.

Correspondence to: Rüdiger Behr (rbehr@dpz.eu)

The copyright of individual parts of the supplement might differ from the CC BY 4.0 License.

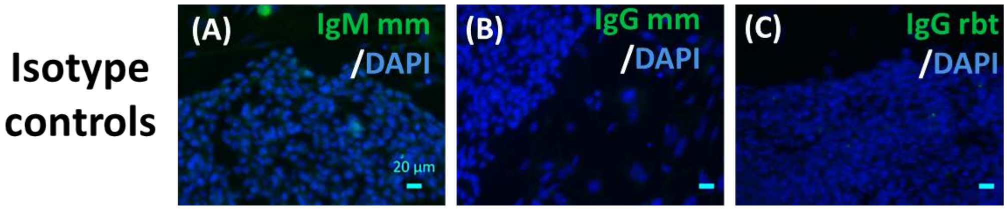

Suppl. Figure 1: **Immunofluorescence isotype control (A), (B), (C)** Isotype controls, merge images FITC+DAPI (scale bar 20µm) **(A)** Isotype control Mouse IgM **(B)** Isotype control Mouse IgG **(C)** Isotype control Rabbit IgG.

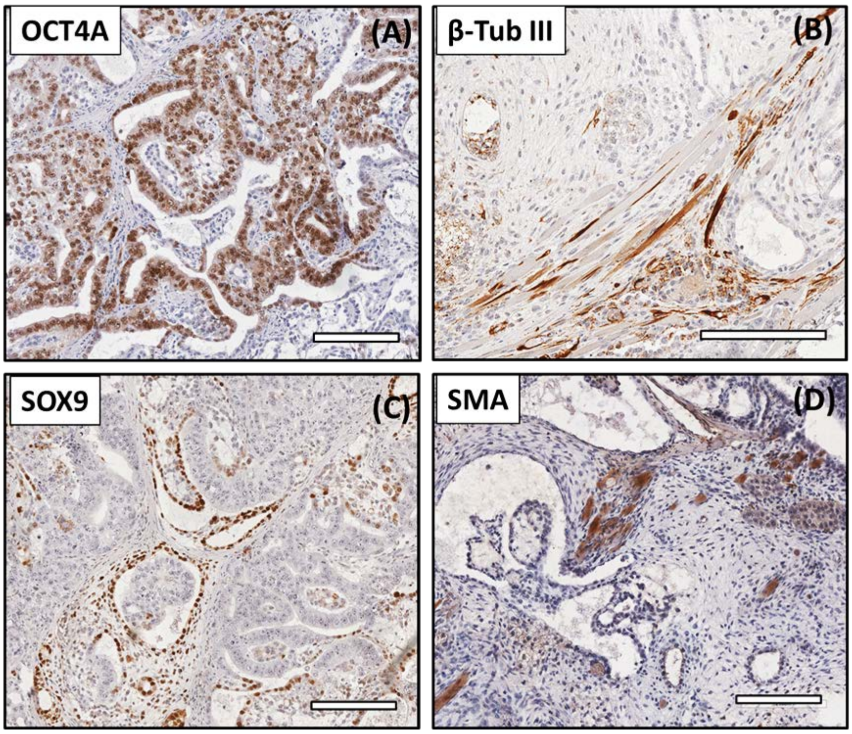

Suppl. Figure 2: Immunohistochemical analysis of DPZ_biPSC 5 teratoma. Tumor tissue analyzed for the expression of SOX9, β-Tubulin III, and smooth muscle actin (SMA), and OCT4A. **(A)** Pluripotency marker OCT4A (POU5F1) staining (bars represent 200 µm). **(B)** Ectodermal β-Tubulin 3 staining (β-Tub III). **(C)** SOX9 staining suggesting endodermal lineage differentiation. **(D)** Mesodermal smooth muscle actin (SMA) staining.
